# Observational Study of Thrombotic Events in a Random Cohort of Hospitalized COVID-19 Patients at a Community-Based Hospital of New York City During the Beginning of the 2020 Pandemic

**DOI:** 10.7759/cureus.18601

**Published:** 2021-10-08

**Authors:** Ruchi Yadav, Beka Aroshidze, Vivek Yadav, Umar Zahid, Apoorva Jayarangaiah, Anjula Gandhi, Vladimir Gotlieb

**Affiliations:** 1 Internal Medicine, Brookdale University Hospital and Medical Center, Brooklyn, USA; 2 Pulmonary and Critical Care, State University of New York Downstate Health Sciences University, New York, USA; 3 Nephrology, Brookdale University Hospital and Medical Center, Brooklyn, USA; 4 Internal Medicine, New York City (NYC) Health and Hospitals/Jacobi Medical Center, Bronx, USA; 5 Hematology/Oncology, Brookdale University Hospital and Medical Center, Brooklyn, USA

**Keywords:** cerebrovascular accidents (cva), deep venous thrombosis (dvt), stroke, pulmonary embolism, myocardial infarction, thrombosis, troponin-i, d-dimer, covid-19

## Abstract

Coronavirus disease 2019 (COVID-19) continues to pose an unprecedented challenge for the entire world and the healthcare system. Different theories have been proposed elucidating the pathophysiological mechanisms attributing to high mortality and morbidity in COVID-19 infection. Out of them, thrombosis and procoagulant state have managed to earn the maximum limelight.

We conducted an observational study based on data from randomly selected 349 hospitalized patients with COVID-19 infection in a community-based hospital in New York City during the first wave of the COVID-19 viral surge in March 2020. The main objective of our study was to assess the risk and occurrence of thrombotic events (both venous and arterial) among the hospitalized patients including the intensive care unit (ICU) and non-ICU admissions with confirmed COVID-19 infection. The primary outcome in our study was defined as the thrombotic events that included myocardial infarction (MI), deep venous thrombosis (DVT), cerebrovascular accidents (CVA), and pulmonary embolism (PE). The study correlated the association of thrombotic events with the level of biomarkers of interest: D-dimer >1000 ng/ml, troponin-I >1 ng/ml, or both. The association of D-dimers and troponin-I with thrombotic events was measured using both univariate and multivariate Cox proportional hazard (PH) regression analysis. Out of a total of 349 patients, 78 patients (22.35%) were found to have elevated biomarkers (D-dimer >1000 ng/ml and/or troponin-I >1 ng/ml) and were categorized as a high-risk group. Eighty-nine patients developed thrombotic complications (evidence of more than one thrombotic event was found in several patients). Two-hundred seventy-one (77.65%) patients had no documentation of thrombosis. The incidence of thrombotic events included myocardial infarction (MI; N=45; 12.8%), cerebrovascular accidents (CVA; N=16; 4.5%), deep venous thrombosis (DVT; N=16; 4.5%), and pulmonary embolism (PE; N=9; 2.57%).

## Introduction

The total number of cases of coronavirus disease 2019 (COVID-19) in the United States of America (USA) since January 21, 2020, was 25,921,703 with 438,035 total deaths at the time of writing this article in April 2020 [1]. The role of D-dimer and troponin-I as markers of thrombosis or procoagulant state in COVID-19 infection has been under investigation since the inception of this disease [2]. Several studies have contributed to elucidating the underlying pathology and correlation of COVID-19 infection with thrombosis [3]. We conducted a retrospective observational study on COVID-19 positive patients, admitted during the first surge of the pandemic in March 2020, including various risk factors such as race, age, gender, body mass index (BMI), comorbidities (diabetes mellitus, hypertension, coronary artery disease), prior use of anticoagulants, use of anticoagulants in current admission, length of stay, ICU admission, and in-patient mortality. D-dimer >1000 ng/ml and troponin-I >1 ng/ml were used as biomarkers for posing a higher risk of thrombosis in COVID-19 infection. The clinical outcome of thrombosis was observed in the form of deep venous thrombosis (DVT), pulmonary embolism (PE), myocardial infarction (MI), cerebrovascular accidents (CVA), or other thrombotic events such as splenic vein thrombosis and portal vein thrombosis. The results of our study data suggested that although the high D-dimer and troponin-I values were associated with the development of thrombotic events, they lacked reliability as conclusive markers. The occurrence of thrombotic disease in patients with D-dimer <1000 ng/ml and/or troponin-I <1 ng/ml indicates that the underlying etiology and pathology of COVID-19 goes beyond the concept of vascular thrombosis and procoagulant state. The use of anticoagulants in established COVID-19 infection, based on the various levels of D-dimer, needs more clinical trials and validations to formulate the standardized protocol

## Materials and methods

Our study included a random selection of 349 patients, aged above 18 years, admitted to a community hospital in New York City, between March 15, 2020, to April 30, 2020. All patients had tested positive for severe acute respiratory syndrome coronavirus 2 (SARS-CoV-2) using a reverse transcriptase-polymerase chain reaction (RT-PCR) of the nasopharyngeal or oropharyngeal swab. The primary outcome in the study was the occurrence of any thrombotic event (both venous and arterial) and its association with the level of the biomarker of interest (D-dimer >1000 ng/ml and/or troponin-I >1 ng/ml). The thrombotic events included MI (EKG findings, cardiac biomarkers, and/or clinical symptoms), DVT (positive venous duplex study), CVA (positive CT and/or MRI of the brain), and PE (positive CT-angiogram of the chest or ventilation/perfusion scan). All findings were confirmed by manual chart review. Additional chart review was performed on specific investigations such as echocardiograms, CT scans, or venous duplex. Diagnoses were made during the inpatient clinical care, as screening for thrombotic events is not a standard method. Most patients received either low-dose prophylactic anticoagulation for the prevention of venous thromboembolism and/or therapeutic anticoagulation. Demographic data included age, sex, race/ethnicity, body mass index (BMI), and data about co-morbidities such as diabetes, coronary artery disease, and hypertension were collected.

The statistical analysis involved non-normally distributed continuous variables that were described as the median and interquartile range (IQR) while mean and standard deviation were used to describe normally distributed continuous variables. The mean of continuous variables was compared using the t-test and Wilcoxon rank test where applicable. Categorical variables were described as number and percentage and were compared using the chi-square test, and Fisher’s exact test was used where the limited data was available. The association of D-dimer and troponin-I with thrombotic events were measured using both univariate and multivariate Cox proportional hazard (PH) regression analysis. Schoenfeld residuals were used to check the assumptions of the Cox PH model and no violations of the assumptions were present.

## Results

The mean age of 349 randomly selected, hospitalized, consecutive COVID-positive patients were 65.1 years (SD 14.44) with 179/349 (51.29%) above 65 years of age. One-hundred sixty-three out of 349 (46.70%) were female and 186/349 (53.30%) were male (Table 1). It was noted that there was no statistically significant difference found between males and females and BMI did not correlate with any higher incidence of thrombotic events. Regarding race, 238/349 (68.19%) were African American patients, 58/349 (16.62%) were Caucasian, 31/349 (8.88%) were Hispanics and 22/349 (6.33%) patients were of unknown ethnicity. Amongst the 349 patients, there were 89 (25.50%) documented cases of thrombotic events and the remaining 271 (77.65%) did not show either the risk or evidence of thrombotic complication. These 89 recorded thrombotic events consisted of MI (50.6%), Stroke (18%), DVT (18%), PE (10%), and others (3.37%) (Table 1). Patients were also categorized into a high-risk group, which was defined as patients with D-dimer >1000 ng/ml and/or troponin-I >1 ng/ml (Table 1), and it is interesting to note that 78/89 (87.64%) of the patients that developed thrombotic events fell into this high-risk group category. Within the high-risk group of patients that developed thrombotic events, 45 (58%) were male and 33 (42%) were female. Additionally, 61/78 (78.21%) of the patients in this high-risk group were African-American, 6/78 (7.69%) were white, 4/78 (5.13%) were Hispanic, and 7/78 (8.97%) were of unknown ethnicity (Table 1). The patients were also divided into three separate ranges of age: 18-44 years (31/249 (8.88%)), 45-65 years (139/349 (39.83%)), and >65 years (179/349 (51.28%)). The patients between 18-44 years had a lower risk of thrombotic events, as 26/271 (9.79%) had no thrombotic events as compared to 5/78 (6.41%) of the high-risk patient group, which developed thrombosis. Meanwhile, age>65 years carried the highest risk of thrombosis as 39/78 (50%) were in the high-risk group vs the 18-44 years category who were only 5/78 (6.41%).

**Table 1:** Detailing all of the relevant patient characteristics and laboratory data HTN- Hypertension, DM- Diabetes mellitus, CAD- Coronary artery disease, DVT- Deep venous thrombosis, PE- Pulmonary embolism, IQR- Interquartile range

Variables	All patients (N=349)	No thrombotic event N=271 (77.65)	Total number of documented thrombotic events (89)						p-value
			High risk of thrombotic events N=78 (22.35)	PE (N=9)	Stroke (N=16)	MI (N=45)	DVT (N=16)	Others (N=3)	
Age, mean (sd)	65.1 (14.44)	64.9 (14.76)	65.7 (13.32)	57.3(9.54)	65.5 (14.23)	66.2 (11.85)	68.9 (16.34)	52.6(6.35)	0.7
Age, n (%)									
18-44	31 (8.88)	26 (9.59)	5 (6.41)	2 (22.22)	2 (12.50)	1 (2.2)	1 (6.25)	0 (0.00)	0.64
45-65	139 (39.83)	105 (38.75)	34 (43.59)	5 (55.56)	7 (43.75)	21 (46.67)	5 (31.25)	3 (100.0)	
>65	179 (51.29)	140 (51.66)	39 (50.00)	2 (22.22)	7 (43.75)	23 (51.11)	10 (62.50)	0 (0.00)	
Sex n (%)									
Male	186 (53.30)	141 (52.03)	45 (57.69)	7 (77.78)	12 (75.00)	25 (55.56)	9 (56.25)	0 (0.00)	0.37
Female	163 (46.70)	130 (47.97)	33 (42.31)	2 (22.22)	4 (25.00)	20 (44.44)	7 (43.75)	3 (100.0)	
BMI, mean (sd) n (%)	30.6 (6.37)	30.2 (6.34)	31.90 (6.37)	32.6 (8.63)	30.5 (6.64)	31.6 (5.79)	33.3 (7.81)	27.8 (3.98)	0.051
Race, n (%)									
African American	238 (68.19)	177 (65.31)	61 (78.21)	6 (66.67)	11 (68.75)	37 (82.22)	12 (75.00)	2 (66.67)	0.029
Hispanic	31 (8.88)	27 (9.96)	4 (5.13)	0 (0.00)	1 (6.25)	3 (6.67)	0 (0.00)	1 (33.33)	
White	58 (16.62)	52 (19.19)	6 (7.69)	1 (11.11)	1 (6.25)	3 (6.67)	2 (12.50)	0 (0.00)	
Unknown	22 (6.30)	15 (5.54)	7 (8.97)	2 (22.22)	3 (16.67)	2 (4.44)	2 (12.50)	0 (0.00)	
Co-morbidities n (%)									
HTN	253 (72.49)	189 (69.74)	64 (82.05)	7 (77.78)	12 (75.00)	38 (54.44)	16 (100.00)	1 (33.33)	0.03
DM	186 (53.30)	140 (51.66)	46 (58.97)	5 (55.56)	9 (56.25)	26 (57.78)	7 (43.75)	2 (66.67)	0.25
CAD	45 (12.89)	25 (9.23)	20 (25.64)	1 (11.11)	4 (25.00)	14 (31.11)	5 (31.25)	3 (100.00)	<0.001
D-dimer, median (IQR)	792 (410-3357)	755 (360-1970)	1891 (587-11233)	1793 (556-25389)	743 (387-3639)	2144 (592-12087)	4594 (690-20729)		0.01
D-dimer, n(%)									
<1000	76 (21.78)	64 (23.62)	12 (15.38)	2 (2.22)	4 (25.00)	6 (13.33)	2 (12.50)	0 (0.00)	0.01
≥1000	95 (27.22)	64 (23.62)	31 (39.74)	3 (33.33)	7 (43.75)	15 (33.33)	9 (56.25)	1 (33.33)	
D-dimer not measured	178 (51.00)	143 (52.77)	35 (44.87)	4 (4.44)	5 (31.25)	24 (53.33)	5 (31.25)	2 (66.67)	
Troponin n (%)									
<1 ng/ml	303 (86.82)	250 (92.25)	53 (67.95)	9 (100.00)	14 (87.50)	22 (48.89)	12 (75.00)	3 (100.00)	<0.001
≥1 ng/ml	29 (8.31)	5 (1.85)	24 (30.77)	0 (0.00)	1 (6.25)	23 (51.11)	4 (25.00)	0 (0.00)	
Troponin not measured	17 (4.87)	16 (5.90)	1 (1.28)	0 (0.00)	1 (6.25)	0 (0.00)	0 (0.00)	0 (0.00)	
Risk for thrombosis n (%)									
High risk (D-dimer ≥ 1000 and/or Troponin ≥ 1 ng/ml)	117 (33.52)	69 (25.46)	48 (61.54)	3 (3.33)	8 (50.00)	31 (68.89)	12 (75.00)	1 (33.33)	<0.001
Low risk (D-dimer < 1000 and/or Troponin < 1 ng/ml)	232 (66.48)	202 (74.54)	30 (38.46)	6 (66.67)	8 (50.00)	14 (31.11)	4 (25.00)	2 (66.67)	
DVT prophylaxis	180 (51.58)	147 (54.24)	33 (42.31)	2 (22.22)	9 (56.25)	19 (42.22)	7 (43.18)	1 (33.33)	0.063
Hx of aspirin/anti-coagulation	84 (24.07)	57 (21.03)	27 (34.62)	3 (3.33)	3 (16.67)	21 (46.67)	5 (31.25)	1 (33.33)	0.013
Received full dose of anti-coagulation	129 (36.96)	91 (33.58)	38 (48.72)	3 (3.33)	3 (18.75)	26 (57.78)	11 (68.75)	1 (33.33)	0.015
Length of stay, median (IQR)	7 (3-10)	6 93-100	8 94-110	8 (7-11)	10 (8.5-12.5)	7 (4-10)	9 (7-13.5)	12 (4-15)	0.005
In-hospital mortality, n (%)	139 (39.83)	104 (38.38)	35 (44.87)	4 (44.44)	7 (46.75)	22 (48.89)	5 (31.25)	0 (0.00)	0.3

Of the comorbidities listed in Table 1, hypertension posed as the greatest contributor to the comorbid condition, with 64/78 (82.05%) of patients belonging to the high-risk group and 54/89 (60.06%) that had developed thrombosis. Forty-five out of 349 (12.89%) patients from the study had underlying coronary artery disease (CAD) and 27/89 (30.33%) had thrombotic events (p<0.001). Patients with mean BMI had 31.9% chances of thrombotic events.

Regarding the criteria to categorize the high-risk patient group, the number of patients that were documented to have troponin-I >1 ng/ml was 29/349 (8.31%), out of which 27 of these patients suffered thrombotic complications. Secondly, the total number of patients that were documented to have D-dimer >1000 ng/ml was found to be 95/349 (27.22%), and 35 of these patients suffered thrombotic complications (35/89; 39.33%). However, patients with D-dimer <1000 ng/ml (71/349; 21.78%) had a 14/89 (15.73%) incidence of thrombotic events. The median (IQR) for the length of stay for all patients was seven days, and longer days of stay were associated with a higher risk of thrombosis. In-hospital mortality was seen more in the thrombotic group (44.87%) vs the non-thrombotic group (38.88%). The patients who were already on anticoagulants had 33/89 (37.07%) episodes of thrombosis, out of which the maximum number developed MI.

Table 2 details the univariate and multivariate hazard ratios of various patient characteristics. The following multivariable adjustments were done: age, sex, ethnicity, coronary artery disease, diabetes mellitus, hypertension, DVT prophylaxis, use of aspirin/anticoagulants, and higher D-dimer levels at the presentation. Troponin-I >1 ng/ml had a hazard ratio of 8.62 (95% CI: 5.21, 12.35; p<0.001) and 7.23 (95% CI: 4.09; 12.79; p<0.001) in the univariate and multivariate analysis, respectively (Table 2). Patients with D-dimer >1000 ng/ml and/or troponin-I >1 ng/ml were calculated to have hazard ratios of 5.55 (95% CI: 3.47,8.87) and 5.10 (95% CI: 3.16, 7.56) in the univariate and multivariate analysis, respectively, with p<0.001 (Table 2).

**Table 2:** Univariate and multivariate hazard ratios of various patient characteristics HTN- Hypertension, DM- Diabetes mellitus, CAD- Coronary artery disease

Variable	Any thrombosis			
	Univariate		Multivariate	
	Hazard ratio (95% CI)	p-value	Hazard ratio (95% CI)	p-value
Age, y				
18-44	1 (Reference)		1 (Reference)	
45-65	1.54 (0.60, 3.95)	0.36	0.57 (0.21, 1.54)	0.26
>65	1.42 (0.58, 3.59)	0.46	0.59 (0.23, 1.58)	0.29
Male sex	1.36 (0.87, 2.14)	0.17	0.91 (0.56, 1.46)	0.69
Race				
African American	1 (Reference)		1 (Reference)	
Hispanic	0.49 (0.18, 1.35)	0.17	0.60 (0.21, 1.7)	0.34
White	0.37 (0.16, 0.850	0.02	0.51 (0.21, 1.21)	0.12
Unknown	1.04 (0.47, 2.29)	0.91	1.29 (0.57, 2.96)	0.53
BMI	1.03 (0.99, 1.06)	0.11	1.02 (0.98, 1.05)	0.35
Co-morbidities				
HTN	1.73 (0.99, 3.08)	0.05	1.49 (0.81, 2.76)	0.19
DM	1.22 (0.78, 1.93)	0.37	1.01 (0.61, 1.64)	0.98
CAD	2.99 (1.80, 5.00)	<0.001	1.24 (0.66, 2.31)	0.49
Troponin				
<1	1 (Reference)		1 (Reference)	
≥1	8.62 (5.21, 12.35)	<0.001	7.23 (4.09, 12.79)	<0.001
Troponin not measured	0.36 (0.05, 2.61)	0.313	0.27 (0.04, 2.14)	0.22
D-dimer				
≤1000	1 (Reference)		1 (Reference)	
>1000	3.59 (1.9, 7.01)	<0.001	2.48 (1.25, 5.19)	0.02
D-dimer not measured	1.39 (0.72, 2.68)	0.94	1.05 (0.53, 2.07)	0.87
Risk for thrombosis				
Low risk (D-dimer < 1000 and/or troponin < 1 ng/ml)	1 (Reference)		1 (Reference)	
High risk (D-dimer ≥ 1000 and/or troponin ≥ 1 ng/ml)	5.55 (3.47, 8.87)	<0.001	5.10 (3.16, 7.56)	<0.001

## Discussion

Wuhan city, the capital of Hubei province in China, became the center of the most deadly outbreak of pneumonia in December 2019. By January 7, 2020, a novel virus was isolated by Chinese scientists from the infected patients and was named SARS-CoV-2, previously called 2019-nCoV, later designated as coronavirus disease 2019 (COVID-19) by WHO [4]. After its first occurrence, the virus crossed all geographical boundaries and caused a pandemic as declared by WHO on March 11, 2020, owing to its high infectivity, ability to get transmitted even in the asymptomatic phase, and relatively low virulence [5].

Many studies have discovered facts regarding the transmission and clinical presentations but less is known about the pathophysiology of COVID-19 infection. It is caused by viral entry and infection in locations of angiotensin-converting enzyme 2 (ACE2) receptor expression, such as pulmonary, gastrointestinal, and possibly kidney tissues, including myocytes and vascular endothelial cells [6]. Thrombosis, both in arterial and venous circulations, is propagated through excessive inflammation, platelet activation, endothelial dysfunction, and stasis [7]. Several known biomarkers of COVID-19 infection are emerging nowadays playing an important role in the diagnostic, treatment, and prognostic fields. The correlation of D-dimer values with the disease severity and as a reliable prognostic marker, in the setting of COVID-19 infection, is a matter of debatable research and study [8]. The optimum cut-off value of D-dimer as >1000 ng/ml has 85% sensitivity, 77% specificity, 54.8% positive predictive value (PPV), and 94% negative predictive value (NPV) [9] in predicting the in-hospital mortality [10]. On the other hand, high values of troponin-I (>1 ng/ml) considered as a marker of myocardial injury pose as an independent predictor of mortality in COVID-19 patients [11].

Our retrospective cohort study showed 22.35% (78/349) of patients belonging to the high-risk groups who had D-dimer >1000 ng/ml and/or troponin-I >1 ng/ml. Overall venous thromboembolism (VTE) rate documented by imaging studies was 28.08% (25/89) (DVT + PE) in our study, which was close to the overall VTE rate of 21% shown in the systematic review and meta-analysis done by Malas et al. recently [3]. The in-patient mortality rate among patients in our study group, with a high risk of TE, was 44.87% (35/78) as compared to 38.80% (104/271) in patients without any thrombotic events. The pooled mortality rate in the Malas study among patients with TE was 23% and 13% among patients with and without TE, respectively [3]. Another study investigated the incidence, time course, laboratory features, and in-hospital outcomes of COVID-19 patients with suspected venous thromboembolism [12] and the result showed 31.9% (44/138) had evidence of VTE.

Some of the interesting findings in our study were that 64 patients in the non-thrombotic group had elevated D-dimer >1000 ng/ml but no evidence of thrombotic events. Seventy-six patients out of 349 (21.78%) had D-dimer <1000 ng/ml; however, 14/89 (15.73%) had thrombotic complications, either arterial or venous. D-dimer values at the time of admission in COVID-19 infection have been associated with a heightened risk of thrombosis although the precise incidence is variable in different studies [7,13]. In both univariate and multivariate analysis (Table 2), D-dimer >1000 ng/ml had a hazard ratio of 3.59 (95% CI; 1.9, 7.01; p<0.001) and 2.48 (95% CI; 1.25, 5.19; p<0.001), respectively. Notably higher values of troponin are associated with more admission to intensive care units with higher in-hospital mortality [14-15]. Early recognition of severe forms of the disease is essential for timely triage of the patients, as treatment in the ICU has become a major challenge [2]. Several factors have been identified, including old age, diabetes, hypertension, obesity, and coronary artery disease that modulate the course of COVID-19 disease [4,16-17]. Porfirio et al. published a case series report that large percentages of severely affected COVID-19 patients suffer clinically significant thrombosis [18]. Our study substantiated the correlation of elevated D-dimer values >1000 with the high risk of thrombosis as 39.74%. Another study based on the New York City health system concluded a 16% incidence of thrombotic events and the independent association of D-dimer with thrombotic events consistent with an early coagulopathy [19].

For further assessment of data, we prepared Table 3 depicting the effect of the use of anticoagulation/DVT prophylaxis on the development of thrombosis. Twenty-eight point eight percent (28.8%; 32/111; p<0.001) of patients who were already on home medications of aspirin and/or anticoagulants developed thrombosis. Patients who received DVT prophylaxis had a 20% (37/180; p<0.53) occurrence of thrombotic events, whereas the ones on full-dose anticoagulation had a 38.7% (43/111; p<0.001) incidence of thrombotic events. More patients need to be studied, based on a large population group, to conclude the use/efficacy of the use of anticoagulation in COVID-19 infections.

**Table 3:** Comparison data of the use of full-dose anticoagulation (AC) and deep venous thrombosis (DVT) prophylaxis on the cumulative result of thrombosis

	On aspirin and/or AC at home (n=111)	Received DVT prophylaxis (n=180)	Received full dose AC (n=111)
Thrombotic event	32	37	43
No thrombotic event	40	110	67
Inconclusive data	39	33	1
P-value	0.001	0.53	0.001

Our study had several limitations, including the selection bias resulting from the hospital-based population, the sample size, possible underdiagnosis of thrombotic phenomena with limited availability of imaging studies due to concerns of transmitting infection or risk of death, and limited testing of D-dimer levels. The type of MI was not confirmed with cardiac catheterization. It was likely that some patients succumbed to the disease so acutely that it led to the underestimation of the burden of thromboembolic events. Numerous methodological limitations in the study prevented it from being definitive, and the lack of screening modalities due to time factors led to the under-diagnosis of the disease.

## Conclusions

Our study group's overall incidence of documented thrombotic events was 25.5% (89/349). Out of a total of 349 patients, 78 patients (22.35%) were found to have both biomarkers elevated (D-dimer >1000 ng/ml and troponin-I >1 ng/ml) and were categorized as a high-risk group. When biomarkers were reviewed individually, patients with D-dimer >1000 ng/ml had a 39.4% risk of thrombosis, and the ones with troponin-I >1 ng/ml had a 24% risk. The overall incidence of in-hospital mortality was higher; 44.87% in the high-risk group as compared to 39.83% in the general population. The lack of prior immunity to COVID-19 has resulted in large numbers of infected patients across the globe and uncertainty regarding the management of the complications that arise in the course of this viral illness. Although D-dimer, sepsis, and microvascular thrombosis are associated with mortality, current data are inconclusive for the use of therapeutic doses of anticoagulation for these findings. Troponin monitoring with serial doses is useful for a short-term follow-up to identify patients who could evolve in a more serious pathological picture than those who could improve through selective cardiac therapies. The true prevalence of thrombosis associated with COVID-19 infection is still under the scanner of various systematic and comprehensive investigation protocols. With the resurgence of the COVID-19 wave, it is imperative to focus on optimal diagnostic and prophylactic strategies to prevent thromboembolic events and potentially improve survival in COVID-19 patients.
